# Modeling WWOX Loss of Function *in vivo*: What Have We Learned?

**DOI:** 10.3389/fonc.2018.00420

**Published:** 2018-10-10

**Authors:** Mayur Tanna, Rami I. Aqeilan

**Affiliations:** ^1^Faculty of Medicine, The Lautenberg Center for Immunology and Cancer Research, Institute for Medical Research, Israel-Canada (IMRIC), Hebrew University of Jerusalem, Jerusalem, Israel; ^2^Department of Cancer Biology & Genetics, Ohio State University Wexner Medical Center, Columbus, OH, United States

**Keywords:** model organisms, knockout, mouse, *Drosophila*, zebrafish, cancer, metabolism, epilepsy

## Abstract

The WW domain–containing oxidoreductase (*WWOX)* gene encompasses a common fragile sites (CFS) known as *FRA16D*, and is implicated in cancer. *WWOX* encodes a 46kDa adaptor protein, which contains two N-terminal WW–domains and a catalytic domain at its C–terminus homologous to short–chain dehydrogenase/reductase (SDR) family proteins. A high sequence conservation of *WWOX* orthologues from insects to rodents and ultimately humans suggest its significant role in physiology and homeostasis. Indeed, data obtained from several animal models including flies, fish, and rodents demonstrate WWOX *in vivo* requirement and that its deregulation results in severe pathological consequences including growth retardation, post–natal lethality, neuropathy, metabolic disorders, and tumorigenesis. Altogether, these findings set WWOX as an essential protein that is necessary to maintain normal cellular/physiological homeostasis. Here, we review and discuss lessons and outcomes learned from modeling loss of WWOX expression *in vivo*.

## Introduction

As a result of perturbed DNA synthesis, chromosomal fragile sites display genomic perturbations in the form of gaps, breaks, constrictions and rearrangements at specific DNA loci on metaphase chromosomes (1–6). The first human chromosomal fragile site was presented in 1965 by Dekaban that was found in an irradiated female patient who received X–rays for the treatment of eczematous dermatitis. These sites harbored two types of chromosomal abnormalities; (a) frequent breaks in the long arm of the chromosome number 9 and (b) moderately increased rate of chromosome–type aberrations occurring at random in all chromosomes (7). Later, in 1970, the name “fragile site” was used to explain recurrent chromosomal breaks on chromosome 16, which followed Mendelian segregation within a large family in a dominant fashion showing linkage of the heptoglobin alpha locus (8). Subsequently, chromosomal fragile sites associated with X–linked mental retardation within different families were reported (9–12). These preliminary reports led to the classification of chromosomal fragile sites as common fragile sites (CFSs) and rare fragile sites, depending on their frequency in the population as well as their inheritance pattern and further subdivided these sites based on the type of inducing chemical (5, 9–14). Ultimately, these early cytogenetic findings intrigued the geneticist community to further explore fragile sites and study their relevance in human diseases.

CFSs are the largest class of chromosomal fragile sites, which represent a component of normal chromosome structure that are observed in all individuals. CFSs are late replicating regions and are known as preferential hotspots for metaphase chromosome breaks and chromosome rearrangements due to partial DNA replication stress (4, 5). In addition, CFSs are reported to be targets of replication stress in preneoplasia as well as sensors for DNA damage (15–18). Normally, CFSs are stable in cell culture settings but are highly susceptible to form chromosome breaks when DNA synthesis is impeded by using low doses of aphidicolin (APH) or hydroxurea (HU) (2, 4). As a consequence, chromosome breaks at 20 CFSs were revealed in lymphocytes treated with low doses of APH causing over 80% of the cytogenetic lesions. *FRA16D*, a genomic region where the *WWOX* gene resides, was noted as one of the most highly expressed CFSs amongst the 20 CFSs identified in human lymphocytes (4). Today, we know that there are about 90 CFSs that are tissue–specific and are induced at a stressor-specific manner [reviewed in (19)]. Based on these findings and others, CFSs were proposed to be involved in chromosome rearrangements observed in cancer (20–23).

*WWOX*, encompassing *FRA16D* at 16q23.2, was cloned and mapped in early 2000 by two research groups simultaneously explaining the significance of this archetypal fragile gene in human cancer (24, 25). The *WWOX* gene spans over 1 Mb of the genomic locus with nine exons separated by large introns encoding 2.2kb mRNA and 1.245kb ORF, which then translates into a 414–amino acid protein product (26). Following these studies, murine *Wwox* (also known as *Wox1)* was cloned suggesting the presence of *WWOX* orthologues in rodents (27). The WWOX protein, as the name suggests, contains two N-terminal WW domains and catalytic domain at its C-terminus homologous to short chain dehydrogenase/reductase (SDR) family proteins. Through its WW1 domain, WWOX interacts with partner proteins harboring proline–tyrosine (PY) motifs and acts as an adaptor protein regulating their localization transactivation, and stability (28, 29). Interestingly, the region of homozygous deletions reported in various malignancies coincides with *FRA16D* where intron eight spans ~750 kb of the *WWOX* gene (25, 30, 31).

Loss of WWOX expression has been described in various tumors including breast, colon, esophageal, gastric, lung, ovarian, and prostate cancers [reviewed in (32)]. Besides its location in a fragile site, WWOX altered expression was also attributed to hypermethylation of its promoter, regulation by microRNAs and posttranslational modifications [reviewed in (26, 33)]. As a result of its dyregulation, several lines of evidence revealed that WWOX affects DNA damage response (DNA repair and apoptosis) (6, 27, 34, 35), cellular metabolism (36–42) and numerous signaling pathways (26, 29, 38, 43–46). Altogether, these observations have led to the generation of animal models mimicking WWOX loss of function *in vivo* to further investigate its physiological significance.

Phylogenetic analysis of the amino acid sequences of WWOX protein reveals that WWOX is an evolutionary conserved protein as a discrete orthologue between insects to humans. The conserved sequences of WW domains and SDR domain in *WWOX* imply its tight regulation across species. All species exhibit distinct sequence relationships of WWOX orthologues through evolution except the closest organism *Caenorhabditis elegans*, which lacks the two N–terminal WW domains but have sequence homology to WWOX SDR domain suggesting common substrate of this putative enzymatic domain independent of its WW domain interactions (47). Indeed, there is a remarkable conservation in sequence, for example, between human *FRA16D*/*WWOX* and mouse *FRA8E1*/*Wwox* both at exon as well as intron levels exhibiting 94% identity along with 96% similarity at protein level (27, 48). Additionally, *FRA16D/WWOX* shares 49% identity and 66% similarity with *Drosophila melanogaste*r homologous protein CG7221/DmWWOX (49).

Several lessons were learned from modeling WWOX perturbations *in vivo* using different animal models facilitating dissecting its significance in physiology and disease states. For example, after a thorough scrutiny of *Wwox* mutant mice, it was proposed that WWOX behaves as a tumor suppressor gene (50–52). Apart from its role in cancer, many reports revealed direct involvement of WWOX in neurological as well as metabolic disorders. Homozygous mutations in *WWOX* are reported in patients with epilepsy and mental retardation (53, 54). Many reports also indicate the role of WWOX in different metabolic disorders including lipid metabolism (55, 56), obesity (57), and type 2 diabetes (58). Increasing lines of evidence are, therefore, suggesting the significant contribution of *WWOX* in human maladies and requiring deeper understanding of WWOX function both at the molecular and cellular levels. In this review article, we describe the knowledge and conclusions learned by modeling loss of WWOX expression *in vivo* using various animal models including mice, rats, *Drosophila* and zebrafish.

## Animal models of *Wwox* ablation

### Mice

Considering the notion that WWOX is frequently lost in human cancers led to generation and characterization of mouse models that depict this ablation to further deepen our understanding of WWOX function in physiology and in pathophysiology. Several mouse models were established and studied.

#### Phenotypic analysis of *Wwox* null mice

The first knockout (KO) mouse model for *Wwox* was established by Aqeilan et al. (50). In this article, the authors altered ~6 kb of DNA sequences within the genomic locus of murine *Wwox* using homologous recombination. This was achieved by replacing exons 2–4 of *Wwox* with a targeting cassette thus generating a *Wwox* mutant allele and later homozygous *Wwox* KO (*Wwox* ^−/−^) mice. These mice were monitored very carefully and phenotyped. While wild type (WT), heterozygous (*Wwox* ^+/−^), and *Wwox* ^−/−^ mice appeared normal in size and were indistinguishable at birth, *Wwox* ^−/−^ pups showed retarded post–natal growth from day 3 and eventually 100% of mice died at 3–4 weeks of age. These KO pups did not exhibit any histological lesions in the main organs but displayed severe metabolic defects and analysis of blood serum chemistry revealed altered levels of carbohydrates, proteins, and lipids hence associating WWOX with cellular metabolism (59). For example, *Wwox* ^−/−^ mice display reduced serum lipid levels (50, 60) as well as impaired expression of steroidogenic enzymes (61).

##### Bone phenotype of Wwox null mice

Severe bone metabolic disease is one of the major and prominent phenotypes observed in *Wwox* ^−/−^ mice. Although no abnormalities in the skeletal patterning was observed, *Wwox* ^−/−^ mice experienced impaired bone metabolism as there was a decrease in the density of trabeculae bone along with thinning of the inner cortex from postnatal day 7 leading to eventual development of smaller limbs in proportion to the body weight of KO pups. This delay in bone formation was the result of cell–autonomous defect in osteoblast differentiation beginning at the mineralisation stage. Reduced trabecular member connectivity and bone surface area in both *Wwox* ^+/−^ and *Wwox* ^−/−^ pups was seen at day 15 in three–dimensional μCT images but the mineral content of trabecular bone in *Wwox* ^+/−^ mice was not significantly changed in comparison to WT. Serum calcium levels were 50% reduced and serum phosphate levels were increased by 20% in *Wwox* ^−/−^ pups indicating a metabolic bone disease. In contrast, *Wwox* ^+/−^ pups did show any altered levels of serum calcium and phosphorous (59)

A physical, functional and molecular link between WWOX and RUNX2, the master regulator of bone differentiation, was later reported. WWOX suppresses transactivation of RUNX2 resulting in repression of many RUNX2 targets. This intimate relationship between WWOX and RUNX2 was also documented in cancer (62–65). In addition to impaired osteoblast differentiation, *Wwox* ^−/−^ mice also exhibited enhanced osteoclast activity (59), though the molecular mechanism of the later is poorly studied. Therefore, it is plausible to conclude that WWOX loss contributes to an osteopenic phenotype and bone metabolic disease. Although this phenotype is very prominent in mice, supporting evidence linking WWOX with osteopenia or osteoporosis in human patients is still lacking. This could be due to the fact that WWOX loss of function due to germline mutations in human patients is postnatal lethal or that patients were not carefully assessed for bone density.

##### Steroidogenesis defect in Wwox null mice

Detailed analysis of the phenotypic abnormalities of *Wwox* ^−/−^ mice also revealed that these pups are born with gonadal abnormalities displaying impaired spermatogenesis (61). A comprehensive study focusing on the defects in the reproductive system of both male and female *Wwox*^−/−^ mice revealed an altered expression of several steroidogenesis related enzymes responsible for this phenotype. Lack of Leydig cells and remarkable reduction of serum testosterone was noted in *Wwox*^−/−^ male mice likely led to the observed testicular hypoplasia. Likewise, *Wwox*^−/−^ female mice have also displayed abnormalities in their reproductive system manifested by impaired theca cells proliferation and small primary follicles resulting in reduced ovary size. Additionally, expression of numerous genes encoding steroid biosynthesis enzymes, such as *Hsd3b6* and *Cyp11a1*, is downregulated in the reproductive organs of *Wwox* ^−/−^ pups when compared with WT and *Wwox* ^+/−^ littermates (61). Altogether, these observations suggest that WWOX has an important role in steroidogenesis and its loss could be associated with infertility though future studies are required to dissect this interesting phenotype.

#### Lessons learnt on WWOX tumor suppressor function from studying *Wwox* mutant mice

##### Wwox null mice

As introduced, altered WWOX expression has been reported in several human malignancies (32). Hence, *Wwox* ablation in mice has been predicted to associate with either tumor initiation or progression. The first *in vivo* evidence associating WWOX with tumor suppression was revealed when juvenile *Wwox* ^−/−^ mice developed femoral neoplastic focal lesions resembling early osteosarcoma (50, 63). These findings were based on histology sectioning through paraffin–embedded bones as well as screening by micro (μ)–CT imaging of intact limbs. These observations paved the way to hypothesize that WWOX deficiency can contribute to osteosarcoma development. Indeed, several reports have shown that WWOX deletion or altered expression is a frequent event in osteosarcoma (63, 64, 66, 67). Since *Wwox* ^−/−^ mice die prematurely, osteosarcoma development could not be followed in adult mice. Nevertheless, a more recent report revealed that somatic ablation of *Wwox* in committed osteoblasts accelerate osteoblastic osteosarcoma formation in *Trp53f/f* mice (68) (also see below).

##### Wwox–heterozygous (Wwox^+/−^) mice

The fact that *Wwox* ablation in mice leads to early postnatal lethality (50) prompted studying WWOX tumor suppressor function using *Wwox*–heterozygous (*Wwox*^+/^^−^) mice. The incidence of spontaneous tumor formation in *Wwox*^+/^^−^ mice was significantly higher than that of WT littermates. Tumors formed in *Wwox*^+/^^−^ mice included development of lung papillary carcinomas and lymphoblastic leukemia, but not osteosarcoma (50). Interestingly, several reports have documented altered WWOX expression in lung cancer (33, 69–71) and lymphomas (72–76) suggesting WWOX as a potential tumor suppressor in these malignancies.

As introduced earlier, perturbations in WWOX is commonly observed in various tumor kinds, other than lung cancer and lymphoma (32). One of the prominent cancer types that display absent or reduced WWOX expression is breast cancer (77–82). Monitoring of *Wwox*^+/^^−^ mice for mammary tumor development in B6/129 mixed genetic background revealed very low incidence (52). These observations led the Aqeilan group to transfer the *Wwox*^+/^^−^ allele onto C3H, a mammary tumor susceptible genetic background. Heterozygous *Wwox*^*C*3*H*+/−^ mice were generated by backcrossing the *Wwox*^+/−^ allele onto C3H pure genetic background mice, for at least 4 generations (51). Monitoring of these mice demonstrated that inactivation of a single *Wwox* allele is associated with increased incidence of mammary tumors. These tumors very much resemble those observed in human basal-like breast cancer (BLBC) with WWOX alteration (83). Poorly differentiated invasive ductal carcinomas and squamous cell carcinomas were observed. Immunohistochemistry staining of hormone receptors: estrogen receptor (ER) and progesterone receptor (PR) were absent/reduced in *Wwox*^*C*3*H*+/−^ mammary tumors. These results, therefore, suggest that heterozygous *Wwox* loss could be sufficient for developing mammary tumors of BLBC-like nature (51). These results were further confirmed in a subsequent recent study by Abdeen and colleagues using a tissue-specific knockout mouse model of *Wwox* (84) (also see below).

##### Chemical–induced tumorigenesis in Wwox–heterozygous (Wwox^+/−^) mice

To further investigate WWOX tumor suppressor function, a number of studies tested susceptibility of *Wwox*^+/^^−^ mice to chemical–induced tumorigenesis. In one study, *Wwox*^+/^^−^ mice were treated with ethyl nitrosourea (ENU)–a mutagen that is commonly used to study tumor spectrum in a given mouse strain. As expected, the incidence of tumor formation, especially lung cancer and lymphoblastic lymphoma, was significantly higher in ENU–treated *Wwox*^+/^^−^ mice in comparison with ENU–treated WT mice (50). In addition, various types of epithelial tumors like liver hemangiomas, chondrosarcomas, fibroadenoma, and squamous cell carcinomas were observed in ENU–treated *Wwox*^+/^^−^ mice. It was also noted that multiplicity of tumors is significantly higher in ENU–treated *Wwox*^+/^^−^mice in comparison with ENU–treated WT mice. Of note, signs of lymphoma aggressiveness were also higher in ENU-treated Wwox^+/−^ mice suggesting that WWOX loss could lead to more advanced tumors (50). These results could suggest that *Wwox*^+/^^−^ mice might be a good model system to study the effects of carcinogens and chemoprevention studies.

In a subsequent study, the susceptibility of loss of one *Wwox* allele on *N* – nitrosomethylbenzylamine (NMBA)–induced forestomach tumors (modeling human esophageal cancer) was assessed. Cohorts of *Wwox*^+/^^−^ and WT mice were treated with NMBA, an environmental carcinogen that is extensively utilized to induce esophageal and forestomach tumors in rodents, and monitored for tumor formation. Consistent with WWOX tumor suppressor function, it was observed that tumor incidence and multiplicity were higher in NMBA–treated *Wwox*^+/^^−^ mice in comparison to NMBA–treated WT littermates (52). These results confirm that perturbations in *WWOX* could be associated with esophegeal tumor formation (85) and further suggest its important tumor suppressor function.

##### Wwox hypomorphic mice

This oncosuppressor function of WWOX was again strengthened by the phenotypes observed in a mouse model generated by the Aldaz group. In late 2007, Ludes–Meyers and co-workers reported the generation of a hypomorphic mouse strain containing mutated *Wwox* alleles using a gene trap approach (86). This mutagenesis resulted in the expression of a *Wwox* splice variant containing intact WW domains but no SDR domain, thus, generating homozygous *Wwox* mutant mouse named as gene trap alleles: *Wwox*^gt/gt^. In these mice, WWOX expression was undetectable in most organs but low levels were found in a minority of tissues, hence, concluding that *Wwox*^gt/gt^ mice are WWOX hypomorphs. Unlike *Wwox* ^−/−^ mice (50), *Wwox*^gt/gt^ mice were viable for up to two years but had decreased survival rate when compared to WT mice. This increased viability phenotype was presumed to be a result of low expression of WWOX in some tissues, which might have been sufficient for postnatal survival of *Wwox*^gt/gt^ mice (86). Consistent with the Aqeilan *Wwox* ^−/−^ mouse model (50), higher incidence of tumor formation was noted in *Wwox*^gt/gt^ mice. *Wwox*^gt/gt^ female mice developed spontaneous B–cell lymphomas that was noted to be invasive in nature with a high degree of infiltration to lymph nodes, pancreas, kidneys, and liver. On the contrary, *Wwox*^gt/gt^ male mice developed lung adenomas as a characteristic of its genetic background. In addition, poorer reproductive capabilities were observed in both young and old *Wwox*^gt/gt^ male mice. The reduced fertility phenotype was predicted to be a consequence of severe degeneration of numerous seminiferous tubules causing premature testicular degeneration, therefore, indicating the important role of WWOX in the development and function of the reproductive system (86). These reports ultimately explained that loss of WWOX expression strongly correlates with tumor formation and aggression, impaired metabolism and defective steroidogenesis, as reported in (50).

#### Conditional *Wwox*–knockout models

*Wwox*-null mice suffer from post–natal lethality, hence restricting studying WWOX functions in adult mice (50). This certainly limits our understanding of WWOX comprehensive function in physiology and homeostasis as well as tumorigenesis. To overcome this limitation, the Aldaz group was the first to generate mice carrying a conditional mutant allele of *Wwox* by targeting exon 1 with LoxP–recombination sites (87). *Wwox*^*lox*/*lox*^ mice were then bred with a general deleter (*EIIA–Cre*) line to develop total *Wwox* KO mice. In agreement with conventional *Wwox* ^−/−^ mice results (50), these conditional *Wwox* KO mice displayed growth retardation and eventually post–natal lethality in comparison to HET and WT mice. The relative weights of multiple organs were significantly reduced whereas brain weight was increased in conditional *Wwox* KO pups consistent with previous reports on *Wwox*–null mice (59). Histology studies of spleen in these conditional *Wwox* KO mice revealed signs of splenic atrophy showing reduced cellularity of red pulp and lymphoid aggregates of white pulp. These mice also displayed signs of leukopenia with lower white blood cell count suggesting impaired hematopoiesis. Interestingly, conditional *Wwox* KO mice had significantly reduced bicarbonate levels in blood, a condition similar to metabolic acidosis. Blood chemistry results of these mice also displayed hypoglycaemia, hypocalcemia as well as had 2–fold higher blood urea nitrogen (BUN) causing kidney failure (87).

In parallel, the Aqeilan group also generated a conditional *Wwox* knockout model (named *Wwox*^*f*/*f*^) by flanking exon 1 of *Wwox* with loxp sites (88). *Wwox*^*f*/*f*^ mice were also crossed with *EIIA–Cre* general deleter mice to generate *Wwox* KO (*Wwox*^Δ/Δ^) mice in all tissues. As expected, *Wwox*^Δ/Δ^ mice displayed significant growth impairments and 100% of mice died by 3–4 weeks of age as a consequence of hypoglycemia and a severe metabolic disorder. Femurs of these mice displayed osteopenic phenotype, defected bone mineralisation process and presence of osteosarcoma–like cells, as observed in previously generated *Wwox* ^−/−^ mice (59). Altogether, both conditional mouse models (87, 88) were very much alike and recapitulated the null phenotype [*Wwox* ^−/−^ mice] (50). Nevertheless, there have been also some inconsistencies in phenotyping these models that shall be further investigated in future studies. Importantly, these models will be beneficial in investigating WWOX function in adult stages (see below).

##### Conditional Wwox ablation in mammary tissue

As mentioned earlier, perturbations in *WWOX* is a common event in breast tumors (32). Therefore, there has been tremendous efforts to uncover whether *WWOX* somatic deletion leads to mammary tumor formation. The Aldaz group generated a mammary-specific knockout of *Wwox* in a mixed 129SV/C57B1/6 genetic background using *BK5–Cre* and *MMTV–Cre* transgenic strains. Unlike the *MMTV–Cre* model which had no effect on mice survival, WWOX homozygous deletion using *BK5–Cre* mice resulted in premature death for unidentified reasons, as reported by the authors (89). Interestingly, both models (*BK5* and *MMTV*) showed aberrant mammary branching morphogenesis displaying defects in branching development, impairment in ductal invasion, and expansion as well as abnormal mammary epithelium development with few ducts having no or reduced number of branching (89). Similar results on mammary development were observed in *Wwox*^*MGE*^^−/−^ mice in B6/129 mixed genetic background, generated by the Aqeilan group using their *Wwox*^*f*/*f*^ mice upon breeding with *MMTV–Cre* transgenic mice (90). Surprisingly, none of these models developed mammary tumors (89, 90) hence questioning initiation role of tumor suppressor WWOX. This observation also led to propose that WWOX is a non–classical tumor suppressor, however this still remains to be proven as it depends on various factors, among which is dependency on cell of origin, combination with other genetic changes or even genetic background of the mouse model utilized as shown previously (51).

Recently, the Aqeilan group reported the first mouse model of somatic deletion of *Wwox* in mammary epithelium with mammary tumors resembling basal–like breast cancer (BLBC) (84). The authors back–crossed *Wwox*^*MGE*^^−/−^ mice onto a mammary tumor susceptible background, *C3H/HeJ* mice, for seven rounds to generate *Wwox*^Δ*MMTV*^. These mice were monitored for the incidence of tumor formation and indeed the majority (~76%) developed mammary tumors with median latency of 270 days whereas no tumors were obtained in WT control littermates. Histological and pathological characterization of these tumors revealed their Grade III invasive ductal carcinoma nature with occasional lung metastasis. The expression of ER and PR was negative in these tumors but ~60% were positive for CK14 hence resembling human BLBC. Further molecular analysis of *Wwox*^Δ*MMTV*^ tumors revealed that somatic loss of WWOX in mammary epithelium results in reduced expression and activity of p53. Interestingly when comparing *Wwox*^Δ*MMTV*^ tumors to those of *Trp53*^Δ*MMTV*^ they cluster very closely indicating that WWOX and p53 cooperate to antagonize breast cancer development. Furthermore, perturbations in *WWOX* and *TP53* co-occur and are correlated with poor survival of breast cancer patients. Altogether, these findings reveal WWOX as an important breast cancer tumor suppressor that regulates p53 function and activity (84)

##### Conditional ablation of Wwox in hepatocytes

Since *Wwox* ^−/−^ mice displayed reduced levels of serum lipids (50) and low expression of steroidogenic enzymes (61), a detailed analysis regarding role of WWOX in lipid metabolism was performed. Microarray analysis of total *Wwox* null mice (*Wwox* ^−/−^) (50) and liver tissue–specific *Wwox* KO (*Wwox* ^*hep*−/−^) mice (60) revealed altered levels of key regulators of high density lipoprotein (HDL) metabolism. Additionally, reduced levels of crucial biomolecules, ApoA−1 and AbcA1, necessary to reverse cholesterol transport and generate nascent HDL particles, were observed in both *Wwox* null and *Wwox* ^*hep*−/−^ mice. Interestingly, *Wwox* ^*hep*−/−^ male mice showed a small decrease in mRNA levels of *AbcA1* but a significant reduction at protein levels. Conversely, unchanged mRNA and protein levels of ABCA1 were observed in *Wwox* ^*hep*−/−^ female mice. This discrepancy of ABCA1 expression at transcriptional as well as translational levels in both the sexes of *Wwox* ^*hep*−/−^ mice was considered to be a gender specific regulation of HDL metabolism. On the contrary, protein levels of ApoA–I were reduced in both *Wwox* ^*hep*−/−^ mice genders. These results were consistent with the observations reported in human disorders of HDL biogenesis suggesting a key role of WWOX in regulating HDL physiology (55). Additionally, older *Wwox* ^*hep*−/−^ female mice exhibited an increase in triglyceride (TG) levels and in very low–density lipoprotein (VLDL)–TG content within serum lipoproteins confirming previously reported association of *Wwox* and TG levels (50). Irrespective of the significant and prominent deregulation of lipid metabolism observed in *Wwox* ^*hep*−/−^ mice, microarray analysis also identified differential expression of several lipid–related canonical pathways specifically revealing the genes involved in cholesterol homeostasis, hydrolysis and biosynthesis of TG and fatty acid between WT and *Wwox* ^*hep*−/−^ mice. Further, network analysis of the microarray data demonstrated decreased HDL metabolism as well as upregulation of *Angptl4, Fasn, Pltp, Gpam, Lipg*, and downregulation of *ApoA-I, Lpl, Insig2*; the key genes involved in several lipid metabolic pathways, hence shedding light on the global and important effects of *Wwox* ablation in liver. Thus, this comprehensive examination of WWOX loss in hepatocytes demonstrated overall deregulation of lipid metabolic pathways along with gender specific regulation of HDL and TG metabolism indicating vital implications of WWOX in atherosclerosis and cardiovascular diseases (60).

In a more recent report, the effect of *Wwox* deletion in hepatocytes was assessed on development of liver cancer and liver regeneration (39). Given that WWOX is frequently altered in liver cancer, Abu–Remaileh and colleagues generated a mouse model with specific targeted deletion of murine *Wwox* alleles in hepatocytes *(Wwox*^Δ*Hep*^*)* and studied consequences on liver biology and development of hepatocellular carcinoma (HCC). Interestingly, *Wwox*^Δ*Hep*^ mice exhibited more potent liver regeneration upon partial hepatectomy. This effect was accompanied with elevated Ki67 staining, a marker of proliferation, and higher levels of c–Myc transcripts, one of the major regulators of liver regeneration. Monitoring *Wwox*^Δ*Hep*^ mice for 2–years didn't reveal increase in spontaneous liver cancer incidence in B6–129 background. Nevertheless, combined ablation of WWOX in hepatocytes and treatment with diethylnitrosamine (DEN), a known liver carcinogen, increased HCC tumor burden and load (39). This outcome was associated with increased levels of HIF1α, WWOX partner that drives aerobic glycolysis (36). WWOX deficiency also resulted in upregulation of HIF1α glycolytic target genes feeding enhanced proliferation. Inhibition of HIF1α activity in DEN–treated *Wwox*^Δ*Hep*^ mice through systemic treatment with digoxin significantly attenuated tumor development suggesting that accelerated HCC development is indeed mediated by increased HIF1α activity. It has been also shown that feeding DEN–treated *Wwox*^Δ*Hep*^ mice with high fat diet synergize to result in enhanced HCC formation. Altogether, these findings indicate that perturbations in *WWOX* could increase the risk of HCC development likely, but not only, through alteration in glucose metabolism (39).

##### Conditional ablation of Wwox in osteoblasts

Osteosarcoma is a highly aggressive and metastatic form of bone cancer that is frequent in adolescents and young adults. As mentioned before, WWOX loss has been associated with human osteosarcoma (63, 64, 66) and some animal models provide support for murine osteosarcoma development (50, 62, 88). Furthermore, WWOX protein is inversely associated with expression and function of RUNX2 (62, 63, 91), which is highly expressed in osteosarcoma and various metastatic cancers (64, 92, 93). To better clarify the role of WWOX in osteosarcoma, the Aqeilan group generated two osteoblast-specific knockout mouse models in which WWOX is ablated in either pre–osteoblasts (*Wwox*^Δ*Osx*1^) or in fully mature osteoblasts (*Wwox*^Δ*Oc*^) (68). Analysis of these mice revealed that *Wwox*^Δ*Osx*1^ mice exhibit a severe defect in osteogenesis, which was associated with induction of p53. Deletion of *Trp53* in *Wwox*^Δ*Osx*1^ mice rescued the osteogenic defect and resulted in the development of high penetrance of poorly differentiated osteosarcomas. The murine phenotype very much resembled human osteosarcoma in different aspects including histology, gene expression resistance to chemotherapy and metastatic behavior (68). Strikingly, it was demonstrated that WWOX might undergo loss of heterozygosity and that it promotes p53 loss of heterozygosity supporting a the emerging role of WWOX in DNA damage response and genomic stability (35, 94) ultimately confirming an intimate relationship between the two tumor suppressors. Importantly, co–occurrence of WWOX and p53 inactivation has been demonstrated as a common event in osteosarcoma (68). Altogether these findings provided the first *in vivo* evidence that WWOX suppresses osteosarcomagenesis through regulating p53 activity.

#### Significance of WWOX in neurological disorders

The high levels of WWOX in the different parts of the brain (95, 96), suggests an indispensable role of WWOX in central nervous system (CNS) homeostasis. Initially, low WWOX expression was reported in the hippocampus of Alzheimer's disease (AD) patients (97) suggesting a plausible role of WWOX in the biology of AD. The Chang group and collaborators have subsequently generated a *Wwox* null mouse by targeting exons 1, 2, 3, and 4 to evaluate physiological significance of WWOX in this neurodegenerative disease. In agreement with previously published data (50), KO mice survived only for about a month (98). Interestingly, it was revealed that homozygous loss of *Wwox* leads to the aggregation of Tau and TPC6AΔ (known to be involved in AD), in brain cortex of juvenile *Wwox* null mice, prior to their death (98). This suggests that WWOX plays a crucial role in inhibiting the aggregation of these plaque forming proteins which cause neurodegenerative diseases (98). Whether specific deletion of *Wwox* in hippocampal or cortical neurons would be associated with or contribute to AD in mice is still unknown.

Numerous subsequent reports documented a number of *WWOX* mutations in different neurological disorders. Indeed, *WWOX* germline mutations were found associated with developmental retardation, ataxia, early onset of epilepsy and intellectual deficiency (53, 54, 99–103). WWOX-mutant patients display a wide range of neurological behaviors ranging from progressive microcephaly, global developmental delay, seizure disorders, bilateral optic atrophy, and spastic quadriplegia in very young infants (~1.5 months) (known as WOREE phenotype, for WWOX–related epileptic encephalopathy), to non–progressive microcephaly and less severe phenotype in adolescence–adult with later onset at 9–12 months (associated with spinocerebellar ataxia type 12 (SCAR12)) (101). This wide range of phenotypic abnormalities could be due to the nature of these mutations. Relatively milder phenotypes seem to originate from missense point mutations (P47T and P47R, G372R), whereas severe manifestations were observed in nonsense mutations (R54^*^, K297^*^, and W335^*^) or partial/complete deletions [reviewed in (38)]. Therefore, WWOX genotypes might correlate with the reported phenotypes, although analysis of more patients would be required to further support this relationship.

WWOX involvement in epilepsy was also documented in animal models. Indeed, *Wwox* gene mutations were first associated with epilepsy and ataxia in mice. Mallaret *et al*. showed that the short–lived *Wwox* KO mouse displays spontaneous and audiogenic seizures (53). This phenotype was also observed in a spontaneous homozygous rat mutation of *Wwox*, (lethal dwarfism, ataxia, and epilepsy) presenting similar phenotype as the *Wwox* KO mice and symptoms similar to mutant *WWOX* patients (104). The molecular function and importance of WWOX in epilepsy and ataxia is largely unknown and is currently under investigation by several labs.

## Rats

The role of WWOX in biology was also documented in rats as an animal model. The phenotype of spontaneously mutated–Lethal dwarfism with epilepsy (*lde*) in rats display severe dwarfism, early post–natal lethality, a high incidence of epileptic seizures, male hypogonadism and the presence of numerous extracellular vacuoles of different sizes in the hippocampus and amygdala of mutant brain (105). To define the genes responsible for the *lde* phenotype, the authors backcrossed *lde/lde* rats with Brown Norway rats to generate *lde/lde* rats with an altered genetic background displaying all the pleotropic phenotypes. Mapping of 1.5–Mbp region of chromosome 19 within *lde* locus revealed a 13 base pair deletion in exon 9 of *Wwox* transcript that caused a frame–shift mutation resulting in the translation of aberrant amino acid sequences at WWOX C–terminus. Considering epileptogenesis as congenital in *lde/lde* rats, these rats were exposed to different sound stimulus resulting in audiogenic seizures that had a pattern beginning with wild running followed by tonic–clonic convolusions. There was no correlation between the age of rat and the incidence of audiogenic seizures. Overall, these results further suggest an important role for WWOX in the CNS as WWOX loss leads to neuronal excitation (104).

WWOX was also described to be a putative target to alleviate symptoms for patients who experience a neuronal injury (106). Here, the authors studied the functional significance of rat *Wwox* and other transcription factors necessary to decide injured neuronal cell fate. Nuclear WWOX levels were rapidly increased in the neurons of sciatic nerve transection model in rats. This induction was accompanied with activation of other known genes involved in neuronal injury such as NF–κB activation and phosphorylation of CREB and JNK1. Eventually, the authors concluded that the elevation of WWOX levels signals the injured neurons to undergo apoptosis through activation of pro-apoptotic pathways (106).

Altogether, *Wwox* manipulation in the rat is associated with important phenotypes highlighting WWOX physiological functions, nevertheless the early death of these rats precludes analysis of adult phenotypes. It is of note that some of the phenotypes reported in the mouse models were not reported or observed in the rat model. This could be related to the approach used in genetic manipulation and/or genetic background.

## Drosophila melanogaster

A high amino acid sequence conservation along with distinct phylogenetic relationship of WWOX across species proposes retention of the major biological functions governed by the *WWOX* gene. The *Drosophila* orthologue of *WWOX* (*DmWwox*) was investigated with an aim to study its involvement in WWOX biological functions. *DmWwox* mutants (*DmWwox*^1^) were therefore generated and characterized (49). The *DmWwox*^1^ mutants were surprisingly viable and exhibited no obvious phenotype. Neverthless, a higher sensitivity and a decrease in the survival rate of *DmWwox*^1^ mutant third instar larvae was observed following dose–dependent irradiation when compared with WT. Protective role of WWOX in *Drosophila* was confirmed when *DmWwox*^1^ mutants rescued the WT phenotype with both *DmWwox* and human *WWOX* constructs. Therefore, based on these results, authors concluded that CFSs and the genes within its vicinity would be involved in protective mechanisms against environmental perturbations (49). Later on, it was reported that background mutation as a result of insertion of *piggyBag* transposon into the second intron of *DmWwox* was responsible for this phenotype. Hence, the authors proposed that a thorough screening of alleles generated by targeted mutagenesis via homologous recombination is mandatory to control the genetic background in *Drosophila* (107). To overcome this drawback and to determine the biological functions of WWOX, a distinct combinatorial approach by using biochemical screening, such as proteomic and microarray screens, as well as genetic analysis to explain functionality of the interactions was performed in *Drosophila*. It was surprising that proteomic analysis did not show any physical interactions of WWOX through its WW domain with PPxY motifs containing proteins. However, functional partners of DmWWOX protein were identified by mass spectrometry and shed light on novel functional characteristics of WWOX contributing to cellular metabolism. The analysis identified significantly altered 13 candidate proteins in *DmWwox* mutants as well as 16 proteins in WWOX ectopically over–expressed adult flies most of which were known to be involved in metabolic pathways. It was striking that two candidate interactors, catalytic enzymes–isocitrate dehydrogenase (*CG6439*) and malate dehydrogenase (*CG7998*) involved in Tri–Carboxylic Acid (TCA) cycle were identified indicating an important contribution of WWOX toward aerobic metabolism. Also several candidates involved in glucose and lipid metabolism were identified. Additionally, enzymes invovled in oxidative stress pathways, such as superoxide dismutase (*Sod*) enzymes were identified as candidate WWOX interactors. This later finding could propose a direct role of the oxidoreductase domain of WWOX in biology of reactive oxygen species (ROS), a known byproduct of oxidative phosphorylation (41).

Using *Drosophila* as a model system, WWOX was also reported to be a modulator of apoptosis via modulation of Caspase-3 and regulation of ROS activity. During *Drosophila* development, ectopic over–expression of low level expression construct for TNFα/Egr caused disruption of repeated ommatidial unit patterning on the eye surface and reduced overall eye size. Introduction of *DmWwox* knock down construct rescued this TNFα/Egr mediated rough eye phenotype as ommatidial patterning across the surface of the eye was restored and the size of the eye was increased. Further, it was shown that WWOX activity is essential for the removal of tumorigenic cells from a developing epithelial tissue (42). Altogether, it is obvious that modeling WWOX loss in *Drosophila* identified conserved WWOX functions in cellular metabolism and apoptosis that has also been reported, at least in part, in mammalian cells (37, 40).

## Zebrafish

The biology of WWOX was also studied in zebrafish as an animal model. Tsuruwaka and co–workers generated antisense morpholinos (MO) and siRNA oligos against *wwox* gene sequence in *Danio rerio* and studied their phenotypes. Embryos harboring genetic manipulated *wwox* were characterized by pericardial edema causing post–natal lethality within a week of age. *Wwox*-deficient zebrafish embryos display growth retardation, as revealed by small eye and head size as well as short body lengths combined with impaired bone development and intracellular Ca^2+^ levels (108). Altogether, *wwox*–mutant zebrafish embryos recapitulate many of the phenotypes of *Wwox*–null rodent models further implicating the conservation of WWOX function across species.

## Summary and future perspective

**Figure 1:**
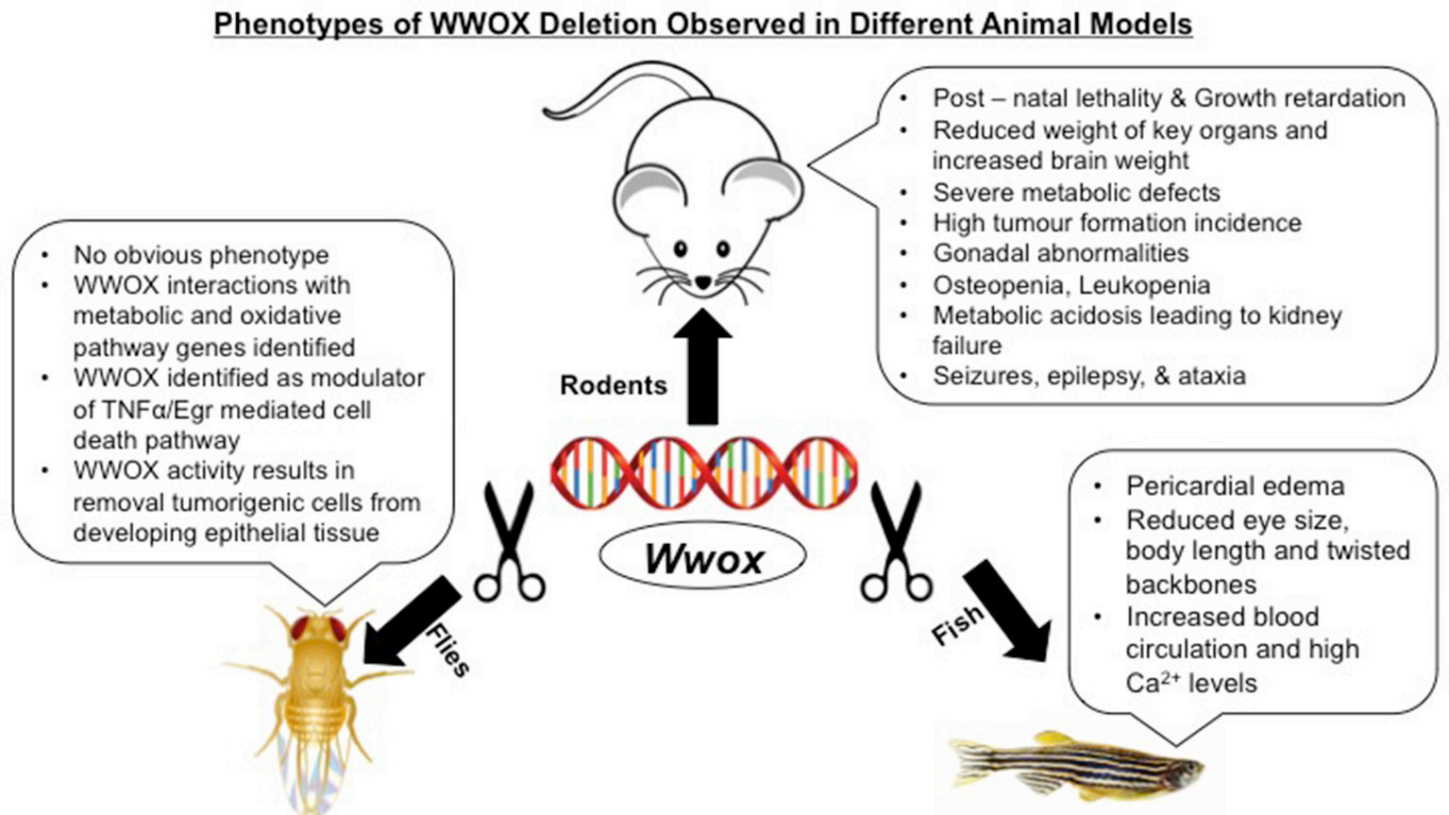
Phenotypes of WWOX deletion observed in different animal models.

Modeling of *WWOX* gene in mice, rats, *Drosophila*, and zebrafish have not only advanced our understanding of its tumor suppressor functions but also have identified its emerging physiological roles in different human pathologies (Figure 1). Targeted ablation of *WWOX* in these established animal models facilitated the characterization of cellular pathways controlled by WWOX and that its deregulation exhibited severe pathological conditions like cancer, CNS related pathologies, metabolic syndromes, and reproductive defects (Table 1).

**Table 1:** Common and unique phenotypes for WWOX animal models.

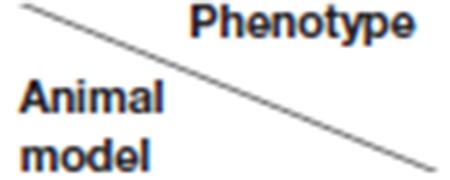	**Post–natal lethality**	**Growth retardation**	**Tumors observed**	**Metabolic defects**	**Reproductivedefects**	**Neurological disorders**	**References**
**Mice**							
*Wwox* ^−/−^	√	√	√ (focal lesions - femurs)	√	√	√	(50)
*Wwox^+/^*^−^	–	–	√ (Chemical +spontaneous)	–	–	–	(50, 52)
*Wwox^*C*3*H*+/^*^−^	–	–	√ (mammary)	–	–	–	(51)
*WWOX*^gt/gt^	↓survival	–	√	–	√	–	(86)
*EIIA–Cre, Wwox^*flox*/*flox*^*	√	√	–	√	√	√	(87)
*Wwox^Δ/Δ^*	√	√	√ (focal lesions – femurs)	√	√	√	(88)
*Wwox^*flox*/*flox*(*Ex*1−4)^*	√	NR	NR	NR	NR	√	(98)
*BK5–Cre, Wwox^*flox*/*flox*^*	√	Mammary gland defect	–	–	–	–	(89)
*MMTV–Cre, Wwox^*flox*/*flox*^*		Mammary gland defect	–	–	–	–	(89, 90)
*Alb – Cre, Wwox^*flox*/*flox*^*	–	–	–	√	–	–	(60)
*Wwox ^Δ*Hep*^*	–	–	√+DEN (Liver)	√	–	–	(39)
*Wwox ^Δ*OSX*1^*	–	–	√+*p53^*f*/*f*^* (osteosarcoma)	–	–	–	(68)
*Wwox ^Δ*MMTV*^*	–	–	√	–	–	–	(84)
**Rats**							
Spontaneously mutated *lde/lde* rat	√	√	–	–	–	√	(104)
***Drosophila melanogaster***							
Mutations generated by homologous recombination	–	–	√	√	–	–	(41, 42)
**Zebrafish**							
Antisense MO and siRNA mediated knockdown	√	√	–	–	–	–	(108)

*NR, stands for not reprorted; -, no phenotypes*.

The phenotypes of *Wwox* null mice include early post–natal lethality, significant growth retardation and dwarfism, severe metabolic defects, osteopenia, and gonadal abnormalities (62, 83, 109). Along with the above mentioned complex phenotypes, *Wwox* null mice also displayed spontaneous and audiogenic seizures as well as showed aggregation of plaque forming protein in the brain cortex indicating that WWOX has an importnat role in the CNS and its loss could lead to epilepsy, ataxia and AD (45, 83). Similar epileptogenic and ataxic phenotype was observed in spontaneously mutated *lde* rats along with male rat hypogonadism (104).

Despite of early post–natal lethality, juvenile *Wwox* null mice phenotype demonstrated the occurrence of lesions resembling osteosarcomas, the first direct evidence of tumor suppressor functions of WWOX (50, 63). Although, this later finding was not detected in all null models but a conserved anti-oncosuppressor function was documented in heterozygous or hypomorph mutant mice (50, 52, 86). Moreover, carcinogen experiments in *Wwox*-heterozygous mutant mice showed higher tumor incidence and multiplicity, compared with control mice, of different tumor types implying WWOX haploinsufficiency (50, 52), a hallamrk of many known tumor suppressors. Conditional *Wwox* KO mouse models recapitulated many of the *Wwox* null phenotypes (87, 88). These conditional mouse models facilitated and will be instrumental in studying the distinct physiological functions of WWOX in a tissue-specific manner. Conditional deletion of *Wwox* in murine mammary epithelium revealed mammary developmental defects suggesting that WWOX expression is indispensable for proper normal breast development (89, 90). More recently, somatic deletion of *Wwox* in mammary epithelium of C3H mice resulted in high penetrance of mammry tumorigenesis that is associated with p53 perturbations (84). Liver tissue-specific conditional *Wwox* KO mouse model revealed WWOX involvement in the complex network of cholesterol homeostasis, HDL and lipoprotein metabolism highlighting a role of WWOX in regulating lipid metabolic pathways and underscoring a therapeutic use of WWOX for atherosclerosis and cardiovascular diseases (60). Somatic *Wwox* deletion in hepatocytes combined with DEN–treatment and/or western diet have been also reported to result in increased HCC incidence further elucidating the tumor suppressor role of WWOX (39).

*Drosophila* models of *WWOX* orthologues identified candidate interactors of WWOX in metabolic as well as oxidative pathways (47). Also, WWOX function for the removal of tumorigenic cells from developing epithelial cells was also reported in the *Drosophila* model (42). Lastly, WWOX ablation within zebrafish embryos displayed a non–cell–autonomous phenotype characterized by the symptoms of pericardial edema, reduced eye size and body length as well as twisted backbones along with high blood circulation and Ca^2+^ levels at ventral–dorsal–posterior regions (108).

Taken together, modeling WWOX in different animal models established WWOX functions in cancer as well as revealed its contribution in metabolic syndromes and neuropathy. These studies identified numerous interacting protein partners of WWOX and indicated new and possible physiological functions (110). The emerging roles of WWOX in DNA damage response (34, 35, 111) and in cellular metabolism (36, 41, 46) could have far reaching effects on the disorders identified in animal models and human patients. Complete understanding of the physiological functions of WWOX is yet under explored and much still remains to be learned about the enzymatic functions of SDR domain. Further characterization of these phenotypic defects, observed both at molecular and cellular levels in WWOX animal models, would be essential for designing novel diagnostic, prognostic, and therapeutic tools.

## Author contributions

All authors listed have made a substantial, direct and intellectual contribution to the work, and approved it for publication.

### Conflict of interest statement

The authors declare that the research was conducted in the absence of any commercial or financial relationships that could be construed as a potential conflict of interest.
